# NEK7 induces lactylation in Alzheimer’s disease to promote pyroptosis in BV-2 cells

**DOI:** 10.1186/s13041-024-01156-9

**Published:** 2024-11-19

**Authors:** Jing Cheng, Hui Zhao

**Affiliations:** Department of Geriatrics, Tongren Hospital, Shanghai Jiao Tong University School of Medicine, 1111 XianXia Road, Shanghai, 200336 China

**Keywords:** Alzheimer’s disease, NEK7, Pyroptosis, Lactylation, Aβ

## Abstract

Alzheimer’s disease (AD), an age-related neurodegenerative disorder, is characterized by irreversible brain tissue degeneration. The amyloid-β (Aβ) cascade hypothesis stands as the predominant paradigm explaining AD pathogenesis. This study aimed to elucidate the mechanisms underlying Aβ-induced pyroptosis in AD. AD models were established using amyloid precursor protein/presenilin 1 (APP/PS1) transgenic mice and Aβ-treated BV-2 cells (5 µM, 24 h). NEK7 expression was evaluated in vitro and in vivo. Cell pyroptosis was assessed before and after NEK7 expression was inhibited in BV-2 cells. Adeno-associated virus (AAV) vectors carrying short hairpin RNA (shRNA) against NEK7 (AAV-sh-NEK7) were administered to mice to knockdown NEK7 in vivo. Spatial learning and memory abilities were evaluated using the Morris water maze test. The interaction between NEK7 and histone H4 lysine 12 lactylation (H4K12la) were then investigated. The results suggested that NEK7 expression was markedly elevated in both in vitro and in vivo AD models. Treatment with Aβ significantly reduced cell viability and enhanced pyroptosis in BV-2 cells; these effects were reversed by inhibiting NEK7. Furthermore, AD mice with NEK7 knockdown exhibited shorter escape latencies and increased time spent in the target quadrant, suggesting that NEK7 inhibition improved cognitive function and memory retention. Mechanistically, Aβ treatment induced histone lactylation in BV-2 cells, and suppression of lactylation attenuated NEK7 transcriptional activity and mRNA levels. In summary, elevated NEK7 expression promoted histone lactylation in BV-2 cells, thereby facilitating pyroptosis. Inhibition of NEK7 conferred protection against Aβ-induced cellular damage and enhanced cognitive performance and memory retention in AD model mice. Collectively, targeting NEK7 represents a potential therapeutic strategy for alleviating AD symptoms.

**Graphical abstract:**

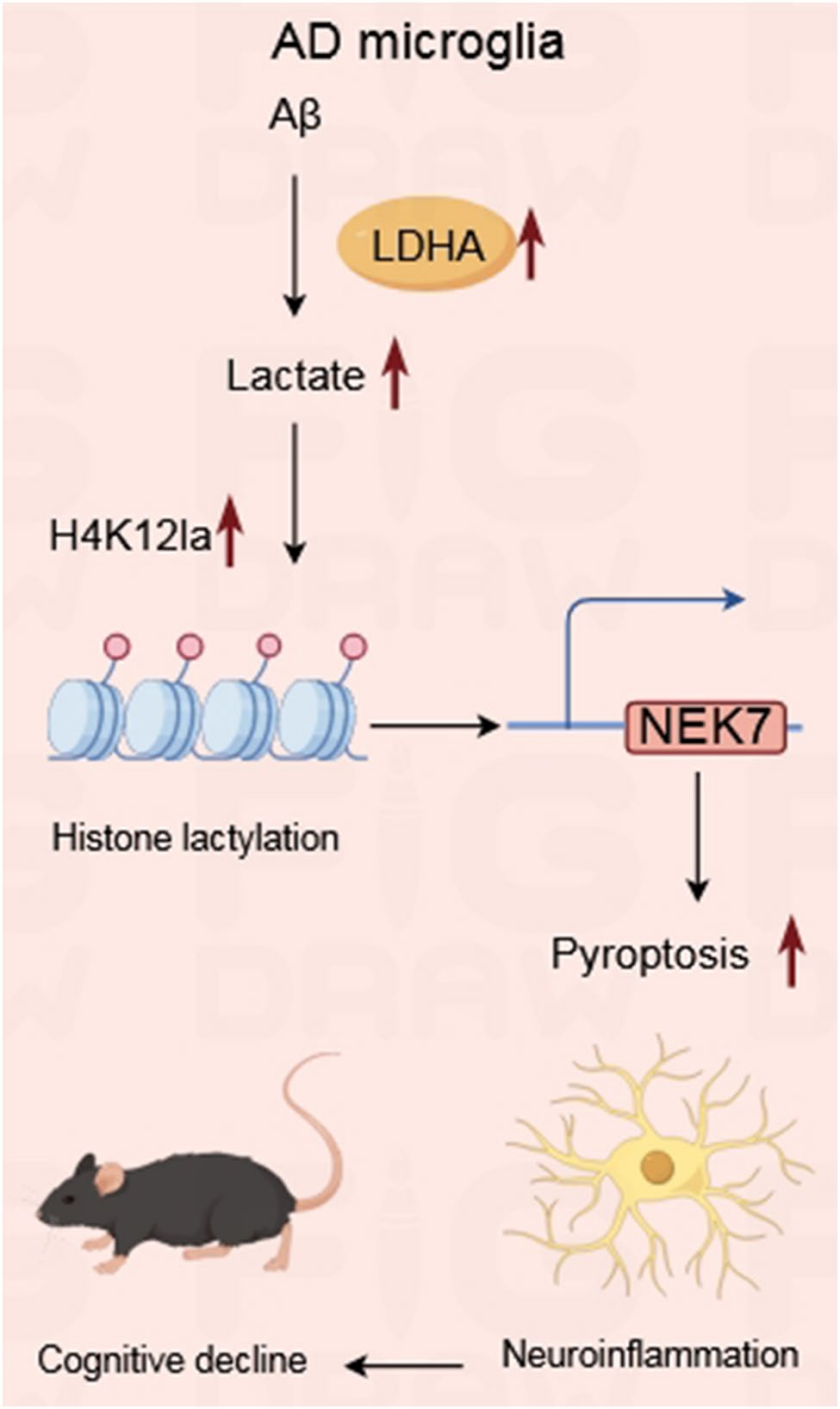

**Supplementary Information:**

The online version contains supplementary material available at 10.1186/s13041-024-01156-9.

## Introduction

Alzheimer’s disease (AD) is an age-related neurodegenerative disorder characterized by an insidious onset. Its primary clinical manifestations include memory impairment, visuospatial deficits, and diminished abstract thinking [1]. The prevalence of AD has steadily increased, making it a major medical and social challenge globally [2]. Pathologically, AD is marked by the presence of senile plaques, formed by amyloid-β (Aβ) deposition, and neurofibrillary tangles, resulting from tau protein hyperphosphorylation [3]. However, the etiology of AD remains complex, and a consensus regarding its pathogenesis has yet to be reached.

It has been established that Aβ can induce pyroptosis, thereby exacerbating the pathological progression of AD [4]. Pyroptosis, a recently identified form of programmed cell death, primarily involves inflammasome-mediated activation of multiple caspase proteases, leading to the cleavage and polymerization of various Gasdermin family members, which subsequently results in the formation of cell membrane pores [5]. Recent studies support the notion that Aβ triggers pyroptosis in microglial cells predominantly through the caspase-1/GSDMD pathway, mediating the release of inflammatory mediators and accelerating the pathological process of AD [5, 6].

NLRP3 is a major pattern recognition receptor involved in pyroptosis and is the most extensively studied inflammasome. Research has demonstrated a close association between AD and the NLRP3 inflammasome [7]. Consequently, elucidating the mechanisms of NLRP3 inflammasome assembly and activation, as well as developing molecular therapeutic strategies targeting downstream components of the inflammasome, could enhance our understanding of AD pathogenesis. NEK7, identified by multiple research groups through various methodologies, is a critical component of the NLRP3 inflammasome, playing a significant role in its activation via potassium efflux [8, 9]. Therefore, it is reasonable to hypothesize that NEK7 may modulate pyroptosis, thereby influencing the progression of AD.

In this study, NEK7 expression was initially detected in brain tissues of AD model mice and in Aβ-induced AD model cells. Subsequently, the regulatory effects of NEK7 on pyroptosis-related factors, amyloid pathology, and memory impairment were examined. Additionally, the underlying mechanisms were investigated. In conclusion, our findings provide a robust experimental basis for understanding the novel mechanism by which NEK7 regulates pyroptosis, thereby affecting the progression of AD.

## Materials and methods

### Bioinformatic analysis

The GSE206478 dataset, downloaded from the GEO database, examines gene expression through RNA sequencing in microglia to elucidate the functional characteristics of genetic variants in AD. Differentially expressed genes (DEGs) between the two groups were analyzed using the online tool Geo2R provided by the GEO database. Genes were screened based on criteria of *P* < 0.05 and ∣log2FC∣≥1.5.

### Animals

The amyloid precursor protein/presenilin 1 (APP/PS1) transgenic AD mouse model used in this study is derived from a C57BL/6J background and harbors the human APP mutation site (K595N/M596L) and the human PS1 mutation site (deletion of the ninth intron). These model mice exhibit learning and memory deficits at 3 months of age, begin to develop age spots at 5 months, and show a significant number of age spots by 12 months, presenting a pathological phenotype similar to that of AD [10]. For this experiment, 7-month-old male APP/PS1 transgenic mice (30–40 g) and their non-transgenic littermates were purchased from the Nanjing University-Nanjing Institute of Biomedicine. Mice were maintained at a room temperature of 22 ± 1 °C under a 12-hour light/dark cycle. All animal procedures were approved by the Animal Experimentation Ethics Committee of Tongren Hospital, Shanghai Jiao Tong University School of Medicine.

Adeno-associated viruses (AAVs) carrying short hairpin RNA (shRNA) targeting NEK7 (AAV-sh-NEK7) and a negative control (AAV-sh-NC) were obtained from GenePharma. Each mouse received an intravenous injection of AAV at a dose of 5 × 10^10 plaque-forming units (PFU) in 20 µL of phosphate-buffered saline (PBS) via the tail vein. The mice were divided into four groups:

- Normal group (*n* = 5): Non-transgenic C57BL/6J mice served as the normal control group.
- AD group (*n* = 5): APP/PS1 transgenic AD mice.
- AD + AAV-sh-NC group (*n* = 65): AD model mice injected with AAV-sh-NC.
- AD + AAV-sh-NEK7 group (*n* = 5): AD model mice injected with AAV-sh-NEK7.

### Morris water maze test

The Morris water maze is a circular pool with a diameter of 120 cm and a height of 60 cm, artificially divided into four quadrants. Different colored reference objects are placed on the walls of the pool in each quadrant. The water maze is equipped with an image acquisition and processing system that automatically records the swimming trajectories of the mice. Parameters such as the time required for the mice to locate the submerged platform, their swimming speed, and other relevant metrics were recorded.

Three days prior to the start of the experiment, the mice were transferred to the behavioral testing room to acclimate to the new environment. Daily handling helped reduce their stress and anxiety. Prior to the commencement of the experiment, the pool was filled to a predetermined depth of approximately 35 cm with water, and food-grade titanium dioxide was added to ensure the water was opaque. A circular platform (approximately 9 cm in diameter) was positioned in the center of the first quadrant. The platform was adjusted to be approximately 1.0 cm above the water surface. The operator then placed the mice on the platform for 60 s to allow them to acclimate to the environment. Subsequently, the mice were released into the water sequentially from the first, second, third, and fourth quadrants (with the mouse’s head directed towards the pool wall) to search for 60 s. Using a long stick to guide them, the mice were allowed to rest on the platform for 20 s after locating it. Adaptive training commenced on Day 1 and continued for a total of three days. Each mouse was given a rest period of 1 h between consecutive water trials. Following the adaptive phase, the water level was raised to submerge the circular platform, positioning it approximately 1.0 cm below the surface. Each mouse was then placed into the water from each of the four quadrants (first, second, third, and fourth) with its head directed towards the pool wall. The recording started when the mouse entered the water and ended when it located the submerged platform and remained there for 5 s. The escape latency was defined as the time taken to find the platform. Additionally, the number of times the mouse crossed the platform position, the swimming distance, and the time spent swimming were used to assess learning and memory abilities.

### Immunofluorescence staining

The mice were anesthetized with sodium pentobarbital (P276000, 100 mg/kg, i.p., AmyJet). Following anesthesia, the brains were collected, and the mice were euthanized by cervical dislocation. The left hemispheres were fixed in 4% paraformaldehyde in PBS at 4 °C overnight and subsequently processed for paraffin embedding. For immunostaining, 5 μm coronal serial sections were deparaffinized and subjected to antigen retrieval using citrate buffer (0.01 M, pH 6.0) at 95 °C for 20 min. The sections were then incubated with 3% hydrogen peroxide (H₂O₂) and washed three times with PBS. Next, the sections were permeabilized and blocked with 10% normal goat serum in 0.3% Triton X-100-PBST for 1 h at room temperature. Following blocking, the sections were incubated with primary antibodies: anti-IBA1 (#17198, Cell Signaling Technology) or anti-NEK7 (MA5-47007, Thermo Fisher). After overnight incubation at 4 °C, the sections were washed with PBS and incubated with the appropriate HRP-labeled secondary antibodies. The immunoreactivity was visualized using diaminobenzidine (DAB). All images were acquired using an Olympus IX73 inverted microscope equipped with a DP80 camera or a Leica TCS SP8 confocal microscope. For each animal, three to seven coronal sections spanning the cortex and hippocampus at different depths were analyzed. Six images were captured from matching areas per section.

### Cell culture and treatment

BV2 microglial cells (American Type Culture Collection) were maintained in Dulbecco’s Modified Eagle Medium (DMEM) supplemented with 10% fetal bovine serum (Hyclone) and penicillin/streptomycin (Beyotime Biotechnology) in a humidified atmosphere with 5% CO₂ at 37 °C. To establish an AD model, BV2 cells cultured in four-well chambers were treated with Aβ_1-42_ (5 µM, Sigma-Aldrich) for 24 h [11, 12]. Small interfering RNAs (siRNAs) targeting NEK7 (si-NEK7), LDHA (si-LDHA), and a control siRNA (si-NC) were obtained from Invitrogen. To knock down NEK7 or LDHA, BV2 cells were transfected with si-NEK7 or si-LDHA using Lipofectamine 2000 reagent (Invitrogen) following the manufacturer’s instructions.

### Western blotting

Western blotting was performed to evaluate protein levels, following established protocols [12, 13]. The antibodies utilized in the Western blotting study included anti-Aβ (sc-28365) from Santa Cruz Biotechnology, anti-NEK7 (ab133514) and anti-caspase-1 (ab138483) from Abcam, anti-GSDMD-N (#39754) from Cell Signaling Technology, anti-L-Lactyl Lysine Rabbit pAb (PTM-1401), anti-L-Lactyl-Histone H4 (Lys12) Rabbit mAb (PTM-1411RM), and Anti-Histone H4 Mouse mAb (PTM-1009) from PTM Biolabs.

### CCK-8 assay

The viability of BV-2 cells in each group was assessed using the Counting Kit-8 (CCK-8) assays (Dojindo) according to the manufacturer’s instructions. Following transfection and different treatments, BV-2 cells were seeded into 96-well plates and incubated with 10 µL of CCK-8 solution. After incubation at 37 °C in a 5% CO₂ atmosphere for 2 h, the absorbance was measured at 450 nm using a microplate reader (BMG Labtech).

### Flow cytometry

Activation of caspase-1 and positive PI staining indicate pyroptosis [14], which was detected using the FAM-FLICA Caspase-1 Assay Kit (ImmunoChemistry Technologies) in this study. Briefly, BV-2 cells (3 × 10^5^) were resuspended in 300 µL of PBS and incubated with 10 µL of FLICA staining solution in the dark for 1 h at 37 °C. The cells were then stained with PI at a concentration of 0.5% (v/v). Detection was performed using a Beckman CytoFLEX flow cytometer, and the results were analyzed with FlowJo software.

### ELISA

The protein levels of IL-1β and IL-18 in the cell supernatants were measured using commercial enzyme-linked immunosorbent assay (ELISA) kits from Sigma-Aldrich, following the manufacturer’s instructions.

### qRT-PCR analysis

Total RNA was extracted using Trizol reagent (Invitrogen). Subsequently, cDNA was synthesized using the Reverse Transcriptase kit (Promega). Gene expression was quantified by real-time PCR using an ABI 7500 Real-Time PCR detection system (Applied Biosystems) with SYBR Green Master Mix (Roche). The cycling conditions consisted of an initial denaturation step at 95 °C for 10 min, followed by 40 cycles of denaturation at 95 °C for 15 s and annealing/extension at 60 °C for 1 min. The specificity of each amplified product was confirmed by analyzing the melting curve of each sample. The relative mRNA expression levels of each gene were normalized to the expression of the housekeeping gene GAPDH. The primer sequences are provided below:

5ʹ-GCTGTCTGCTATATGAGATGGC-3ʹ (NEK7-F) and 5ʹ-CCGAATAGTGATCTGACGGGAG-3ʹ (NEK7-R); 5ʹ-CAAAGACTACTGTGTAACTGCGA-3ʹ (LDHA-F) and 5ʹ-TGGACTGTACTTGACAATGTTGG-3ʹ (LDHA-R); 5′-GTCGCCAGCCGAGCC-3′ (GAPDH-F) and 5′-TGAAGGGGTCATTGATGGCA-3′ (GAPDH-R).

### Chromatin immunoprecipitation PCR (ChIP-PCR)

BV-2 cells were cross-linked with 1% formaldehyde, and the genomic DNA was sheared to an average fragment size of 400 bp. Immunoprecipitation was performed using antibodies specific to H4K12la. The ChIP-PCR primers were designed to amplify the promoter regions containing potential H4K12la binding sites within the NEK7 gene. A positive control antibody (RNA polymerase II) and a negative control non-immune IgG were used to validate the efficacy of the kit reagents (P-2025-48, Epigentek Group). The immunoprecipitated DNA was then purified, released, and eluted. The eluted DNA was used for subsequent PCR analysis.

### NEK7 transcriptional activity determination

Mutated NEK7 luciferase reporter vectors were transfected into BV-2 cells with or without LDHA knockdown, followed by lactate treatment (25 µM, Sigma-Aldrich) [15]. The cells were then lysed, and luciferase activity was measured using a Promega luciferase assay kit (Promega) and a microplate reader (Bio-Rad).

### Statistical analysis

Normally distributed data were compared using Student’s t-test or one-way analysis of variance (one-way ANOVA). Results are presented as the mean ± SD. Statistical significance was set at *p* < 0.05p < 0.05. All statistical analyses were conducted using IBM SPSS 22.0 software.

## Results

### High expression of NEK7 is related to AD

**Fig. 1:**
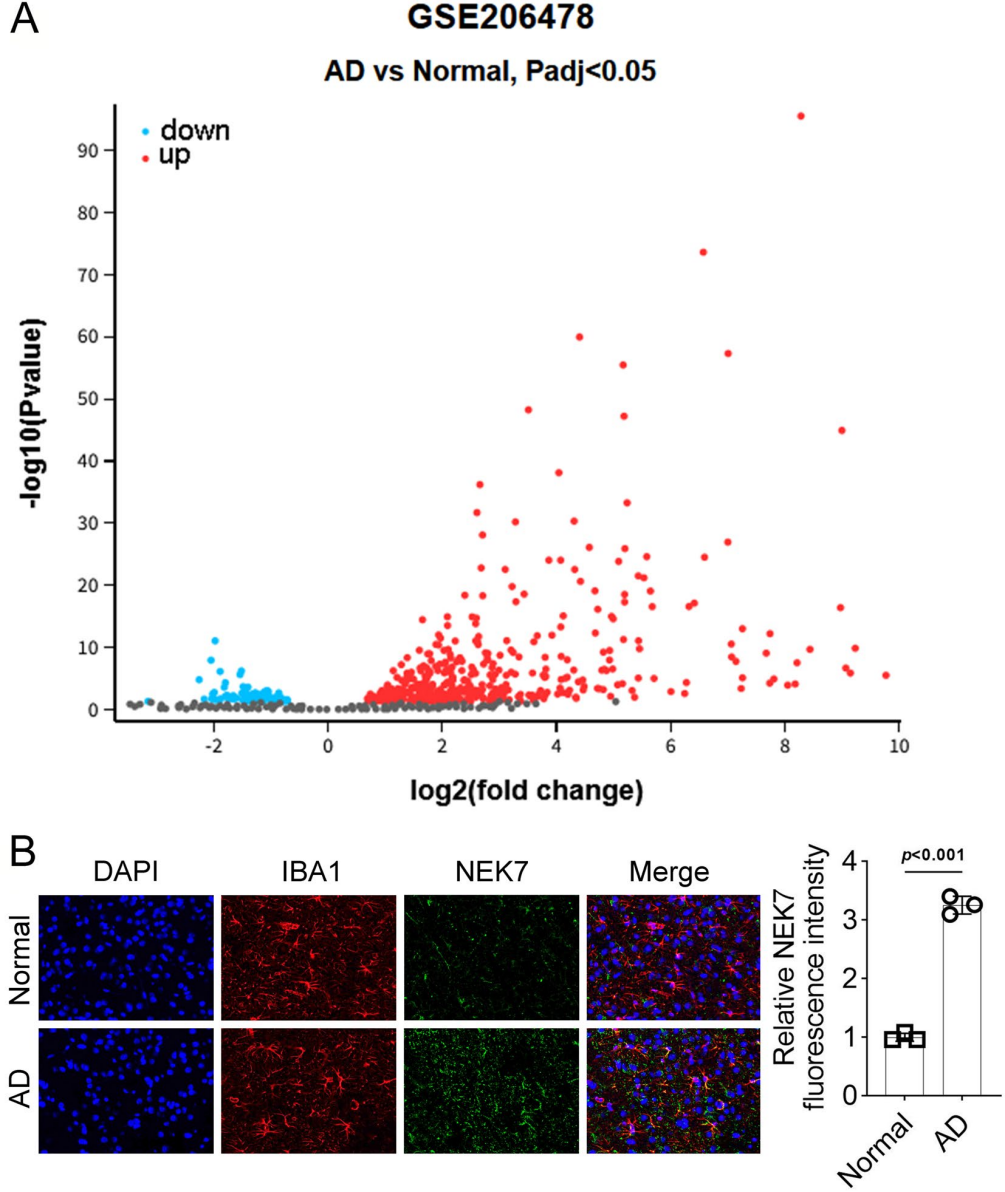
NEK7 is highly expressed in brain tissues of mice with AD. (**A**). Volcano plot illustrating differentially expressed genes in AD identified through bioinformatic analysis. (**B**). Protein expression of NEK7 and IBA1 in brain tissues of AD mice evaluated by immunofluorescence staining

Firstly, bioinformatic analysis identified aberrantly high NEK7 expression in AD (Fig. 1A). Subsequently, the expression of NEK7 in brain tissues from both normal and AD model mice was evaluated using immunofluorescence staining. IBA1, a calcium-binding protein specifically expressed in microglia and commonly used as a marker [16], was co-stained with NEK7. The immunofluorescence staining results revealed that the fluorescence signals of IBA1 and NEK7 were bright and uniform (Fig. 1B, *p* < 0.001).

**Fig. 2:**
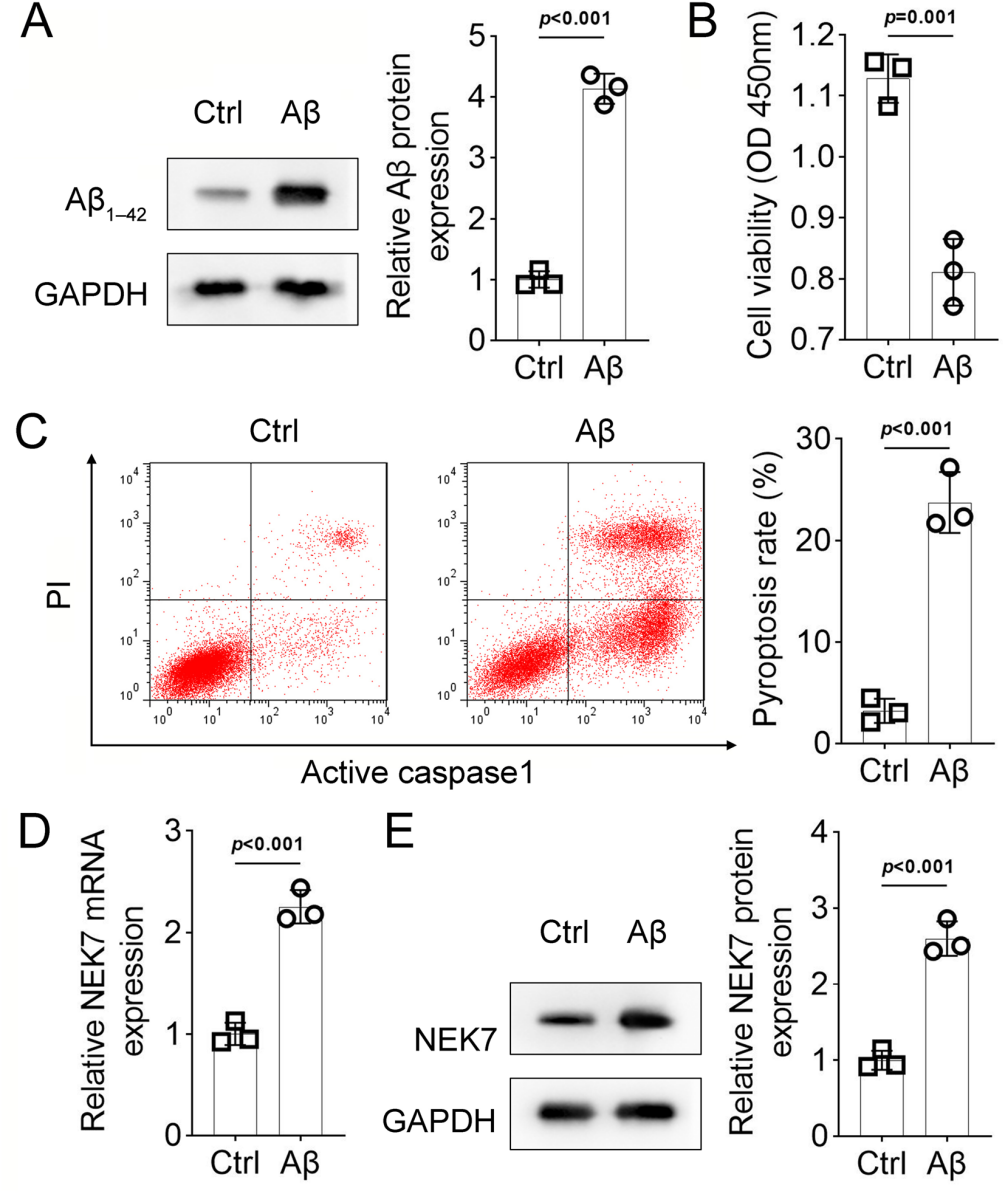
The effects of Aβ on BV-2 cells. (**A**). Aβ deposition in BV-2 cells assessed by Western blotting. (**B**). Cell viability of BV-2 cells under Aβ treatment determined by CCK-8 assay. (**C**). Pyroptosis detection using the FAM-FLICA Caspase-1 Assay Kit. (**D**). mRNA expression of NEK7 in BV-2 cells under Aβ treatment evaluated by qRT-PCR. (**E**). Protein expression of NEK7 in BV-2 cells under Aβ treatment evaluated by Western blotting

Next, the regulatory functions of NEK7 in AD model cells were investigated. Chronic deposition of Aβ stimulates the persistent activation of microglial cells in AD [17]. As shown in Fig. 2A, Aβ deposition was significantly induced in Aβ1-42-treated BV-2 cells (Fig. 2A, *p* < 0.001). Treatment with Aβ prominently inhibited cell viability (Fig. 2B, *p* = 0.001) and increased cell pyroptosis (Fig. 2C, *p* < 0.001). Additionally, both the mRNA (Fig. 2D, *p* < 0.001) and protein (Fig. 2E, *p* < 0.001) expression levels of NEK7 were upregulated by Aβ treatment.

### Effects of inhibition of NEK7 in AD models

**Fig. 3:**
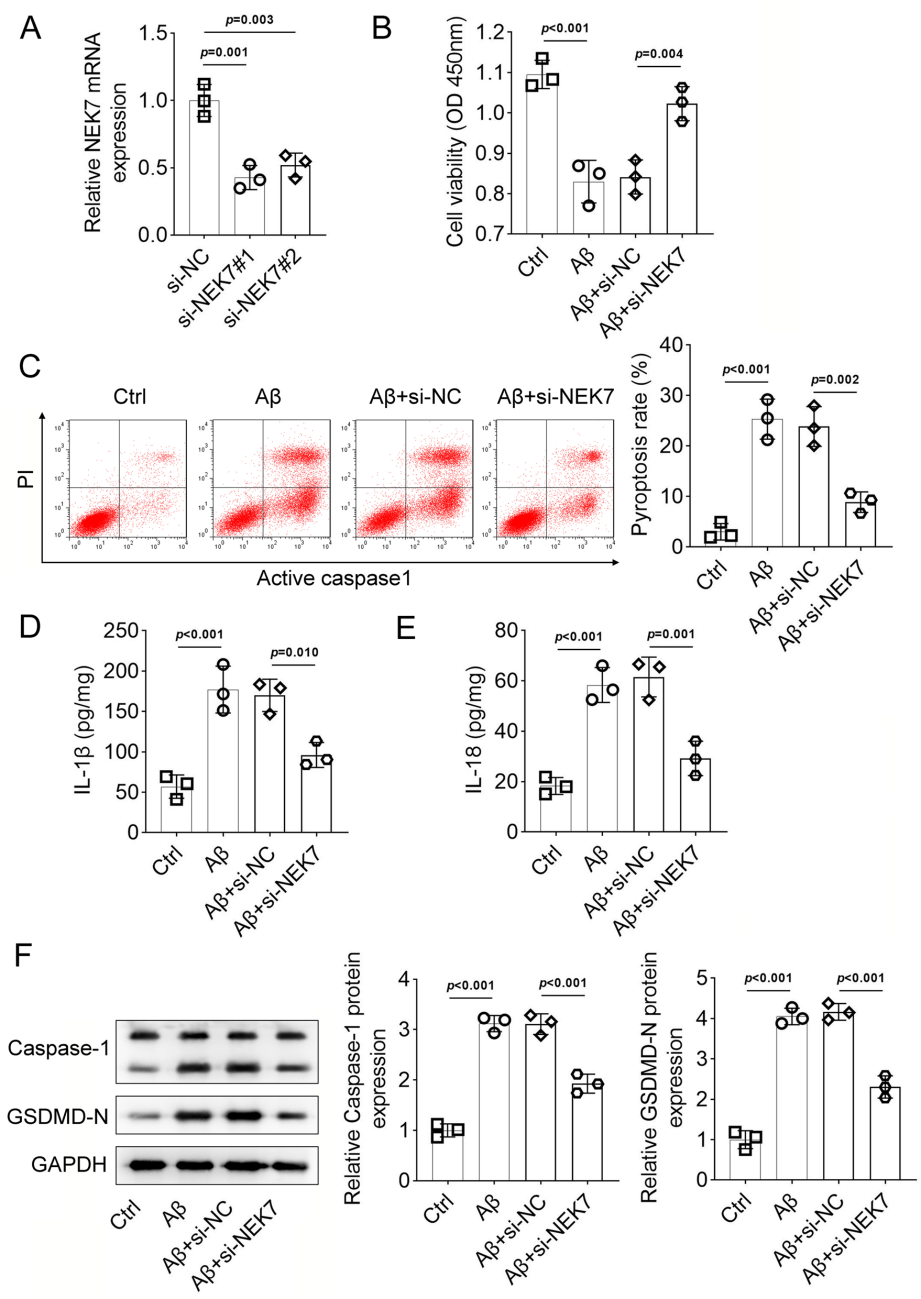
The effects of NEK7 inhibition on BV-2 cells. (**A**). mRNA expression of NEK7 in BV-2 cells transfected with si-NEK7. (**B**). Cell viability of BV-2 cells transfected with si-NEK7 under Aβ treatment determined by CCK-8 assay. (**C**). Pyroptosis detection using the FAM-FLICA Caspase-1 Assay Kit. (**D-E**). Protein levels of IL-1β and IL-18 in BV-2 cells transfected with si-NEK7 under Aβ treatment evaluated by ELISA. (**F**). Protein expression of caspase-1 and GSDMD-N in BV-2 cells transfected with si-NEK7 under Aβ treatment evaluated by Western blotting

The effects of NEK7 were then investigated both in vitro and in vivo. NEK7 was successfully downregulated in BV-2 cells (Fig. 3A, *p* < 0.01). The decreased cell viability and increased cell pyroptosis induced by Aβ were significantly reversed by the inhibition of NEK7 (Fig. 3B and C, *p* < 0.01). Additionally, the increase in IL-1β and IL-18 concentrations caused by Aβ treatment was also reduced by NEK7 knockdown (Fig. 3D and E, *p* < 0.01). Western blotting results showed that the expression of pyroptosis-related proteins induced by Aβ was downregulated by si-NEK7 (Fig. 3F, *p* < 0.001).

**Fig. 4:**
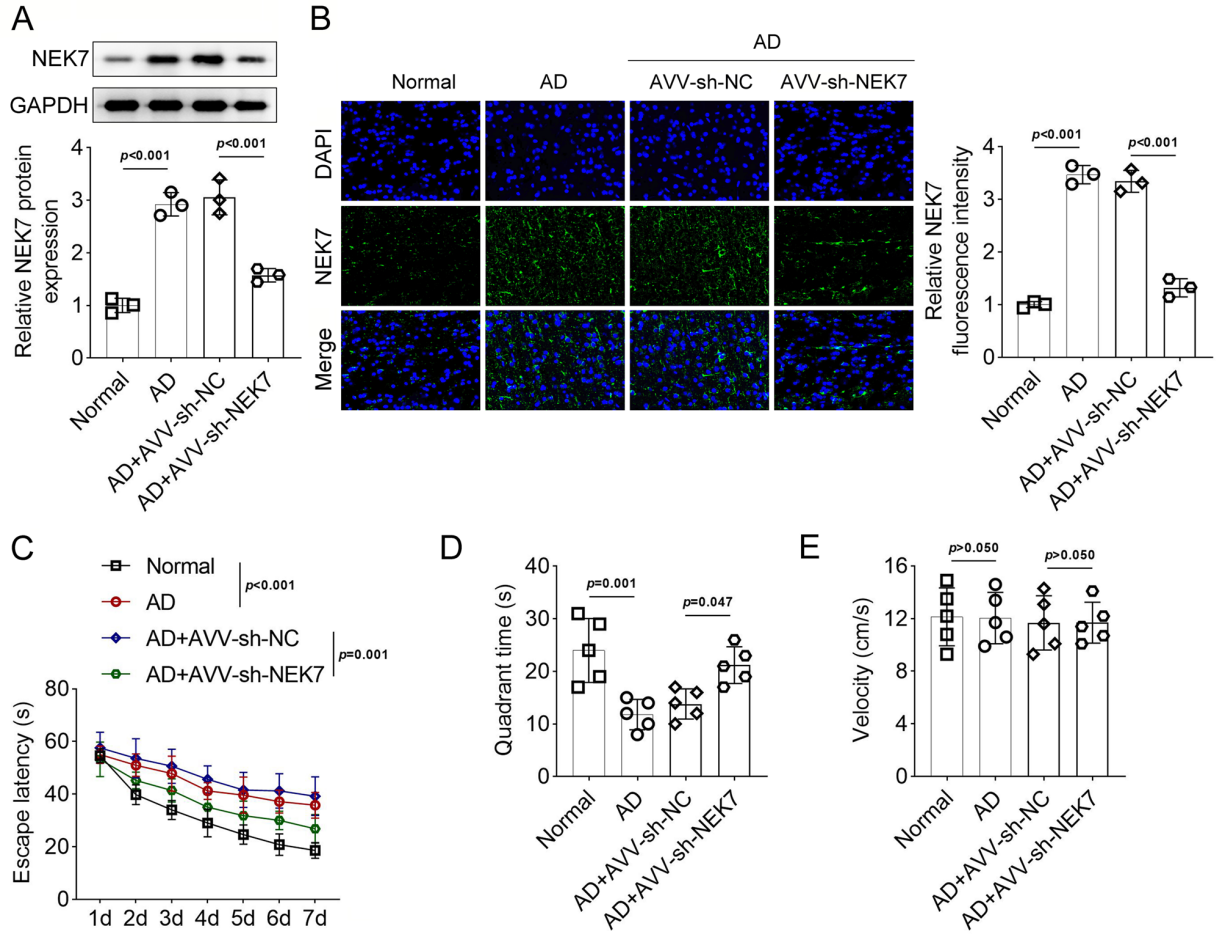
The effects of NEK7 inhibition on AD model mice. (**A**) Protein expression of NEK7 in brain tissues of AD mice with or without sh-NEK7 injection evaluated by Western blotting. (**B**) Protein expression of NEK7 in brain tissues of AD mice with or without sh-NEK7 injection evaluated by immunofluorescence staining. (**C-E**). Morris water maze test for AD model mice with or without sh-NEK7 injection, showing differences in escape latency, quadrant time, and swim velocity during a 7-day training period

Subsequently, NEK7 was downregulated in AD mice, as evidenced by Western blotting (Fig. 4A, *p* < 0.001) and immunohistochemistry (Fig. 4B, *p* < 0.001) results. The escape latency of normal mice decreased over time, whereas that of AD mice increased significantly compared to the normal group (Fig. 4C, *p* < 0.001), indicating a weakening of learning and memory retention abilities. Furthermore, the time spent in the target quadrant was significantly lower in the AD group compared to the normal group (Fig. 4D, *p* < 0.05). Inhibition of NEK7 prominently decreased the escape latency and increased the time spent in the target quadrant, suggesting that NEK7 knockdown alleviated AD progression. The swim velocity did not differ significantly among the four groups (Fig. 4E, *p* > 0.05), indicating no differences in neuromotor function. Taken together, these results suggest that inhibition of NEK7 protects BV-2 cells from Aβ-induced injury and improves learning and memory retention in AD model mice.

### Aβ treatment induced lactylation to promote NEK7 activity

**Fig. 5:**
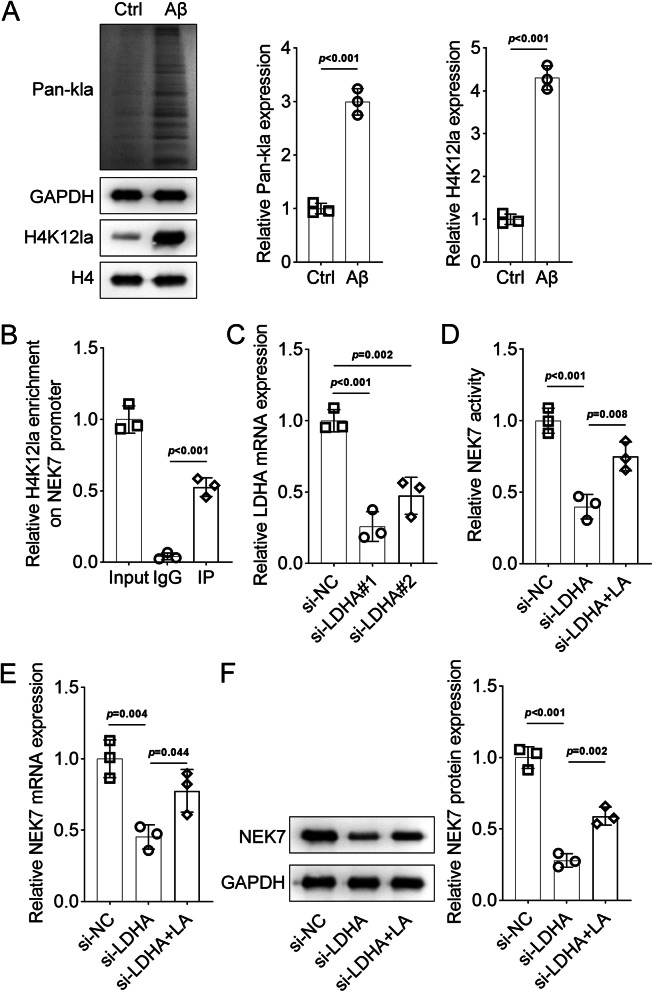
Lactylation of BV-2 cells is related to NEK7. (**A**) Protein levels of lactylation under Aβ treatment evaluated by Western blotting. (**B**) Interaction between the NEK7 promoter and H4K12la assessed by ChIP-PCR. (**C**) mRNA expression of NEK7 in BV-2 cells transfected with si-LDHA. (**D**) NEK7 transcriptional activity in BV-2 cells transfected with si-LDHA. (**E**) mRNA expression of NEK7 in BV-2 cells transfected with si-LDHA evaluated by qRT-PCR. (**F**) Protein expression of NEK7 in BV-2 cells transfected with si-LDHA evaluated by Western blotting

As previously reported, H4K12la levels are elevated in microglia adjacent to Aβ plaques. This lactate-dependent histone modification is enriched at the promoters of glycolytic genes and activates transcription, thereby increasing glycolytic activity [18]. Therefore, we hypothesized that H4K12la may promote NEK7 activity, contributing to its upregulation in AD. As shown in Fig. 5A, Aβ treatment induced both total lactylation and H4K12la levels (Fig. 5A, *p* < 0.001). ChIP-PCR analysis demonstrated that the level of H4K12la antibody targeting the NEK7 promoter was significantly increased compared to that of IgG (Fig. 5B, *p* < 0.001), suggesting that H4K12la may activate NEK7 expression by targeting its promoter. LDHA, which mediates histone lactylation [19], was downregulated in BV-2 cells (Fig. 5C, *p* < 0.01). Inhibition of LDHA significantly decreased the transcriptional activity of NEK7, and lactate treatment partially restored this activity (Fig. 5D, *p* < 0.01). Concurrently, the mRNA and protein expression levels of NEK7 were decreased by LDHA knockdown but increased by lactate treatment (Fig. 5E and F, *p* < 0.05).

## Discussion

The Aβ cascade hypothesis is one of the classical pathogeneses of AD. Aβ is formed through the hydrolysis of APP by β-secretase and γ-secretase [20]. The dynamic balance of Aβ production and degradation can be disrupted by external toxins, infections, or mutations in the APP gene, leading to excessive aggregation of Aβ, which induces pyroptosis and triggers inflammation [21]. Studies have shown that abnormal deposition of Aβ can overactivate microglia, leading to the production of numerous inflammatory factors [22]. Our data demonstrated that Aβ treatment significantly induced Aβ production and promoted the secretion of IL-1β and IL-18, thereby facilitating microglial pyroptosis.

As previously reported, NLRP3 is predominantly expressed in microglia within the central nervous system [23]. NLRP3 inhibitors have been shown to reduce the overactivation of microglia in AD transgenic mice, thereby inhibiting inflammation and pyroptosis [24]. NLRP3 may impair microglia-mediated phagocytic clearance of Aβ, leading to Aβ deposition [25]. Reduced caspase-1 expression has been detected in NLRP3 knockout mice, alongside diminished Aβ aggregation, suggesting that overactivated NLRP3 accelerates the pathological progression of AD [26]. This finding aligns with the work of Heneka et al., who observed increased NLRP3 expression in the microglia of AD mice. Under the influence of Aβ, caspase-1 was activated, and the secretion of IL-1β and IL-18 was induced, thereby enhancing the inflammatory response in brain tissue [24]. Moreover, the P2 × 7 receptor is an ATP-gated ion channel primarily present in immune cells and predominantly expressed on the surface of microglia in brain tissue. Increased expression of P2 × 7 in the brain tissue of AD mice was primarily localized in microglia surrounding aged plaques, suggesting that Aβ may activate the NLRP3 inflammasome through the P2 × 7 receptor expressed on the surface of microglia, thereby inducing pyroptosis in caspase-1/GSDMD-dependent cells [27]. NEK7 is a highly conserved serine/threonine kinase essential for mitosis. It is widely expressed in various tissues and cells and is involved in cellular periodic changes and the regulation of multiple biological processes in vivo [28]. Additionally, NEK7 is a central molecule activated by the NLRP3 inflammasome and is typically less active under normal growth conditions [29]. Upon stimulation, the activated NEK7 protein binds to the leucine-rich repeat (LRR) sequence of the NLRP3 protein independently of its kinase activity, mediating the assembly and activation of the NLRP3 inflammasome. The activated inflammasome further induces an inflammatory response in the body, leading to tissue damage and dysfunction [30]. Therefore, we speculated that NEK7 may regulate pyroptosis in AD. Our data suggested that NEK7 expression was upregulated in AD models, and inhibition of NEK7 significantly suppressed Aβ-induced pyroptosis, contributing to improvements in learning ability and memory retention in AD model mice.

Interestingly, H4K12la levels are elevated in microglia adjacent to Aβ plaques, exacerbating microglial dysfunction, as reported by the team of Pan RY [18]. Therefore, we speculated that NEK7 may participate in this regulatory loop. Our data suggested that Aβ treatment induced both total lactylation and H4K12la levels. H4K12la may activate NEK7 expression by targeting its promoter. Inhibition of lactylation significantly decreased the transcriptional activity of NEK7. Thus, Aβ treatment appears to induce the lactylation of histone H4 lysine 12, promoting NEK7 transcriptional activity, which in turn promotes pyroptosis and the progression of AD.

Animal experiments have small sample sizes and may not capture diversity within a population, such as differences between animals of different sex, age, or genetic background. The confidence intervals for the results may be wide, leading to increased uncertainty in the conclusions. Therefore, the sample size should be further expanded in future studies to verify the conclusions of this study. Furthermore, it is of significance to conduct to alternative experiments (such as Y maze, novel object recognition test and passive avoidance test) to demonstrate the findings of Morris water maze.

## Conclusion

In conclusion, our research indicates that inhibition of NEK7 suppresses microglial pyroptosis and enhances learning ability and memory retention in AD model mice. Moreover, inhibiting the lactylation of H4K12la can reduce NEK7 transcriptional activity, thereby downregulating NEK7 expression.

## Electronic supplementary material

Below is the link to the electronic supplementary material.

Supplementary Material 1

Supplementary Material 2

## Data Availability

The datasets used and/or analyzed during the current study are available from the corresponding author on reasonable request.
