# Supplementary material for: NEK7 induces lactylation in Alzheimer’s disease to promote pyroptosis in BV-2 cells
Source: Mol Brain. 2024 Nov 19;17:81. doi: 10.1186/s13041-024-01156-9 (PMC11577724; doi:10.1186/s13041-024-01156-9)
Supplement: Supplementary file 1 — Supplementary Material 1 [file 13041_2024_1156_MOESM1_ESM.docx]

Figure 1

| 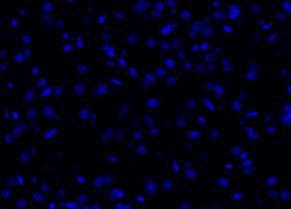 | 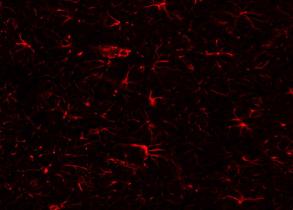 | 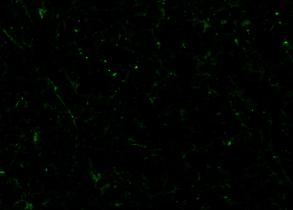 | 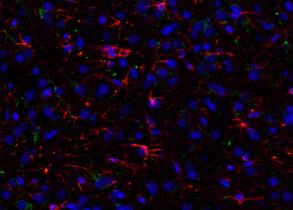 |
| --- | --- | --- | --- |
| 1(1) | 1(2) | 1(3) | 1(4) |
| 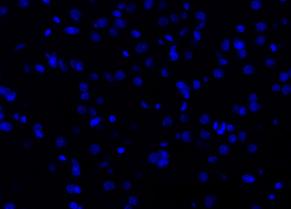 | 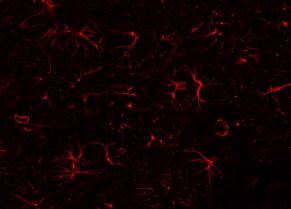 | 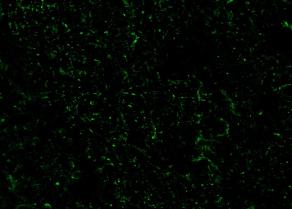 | 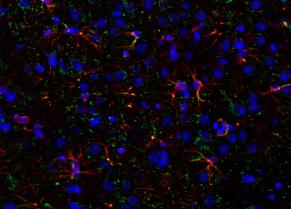 |
| 2(1) | 2(2) | 2(3) | 2(4) |

Figure 2A

| 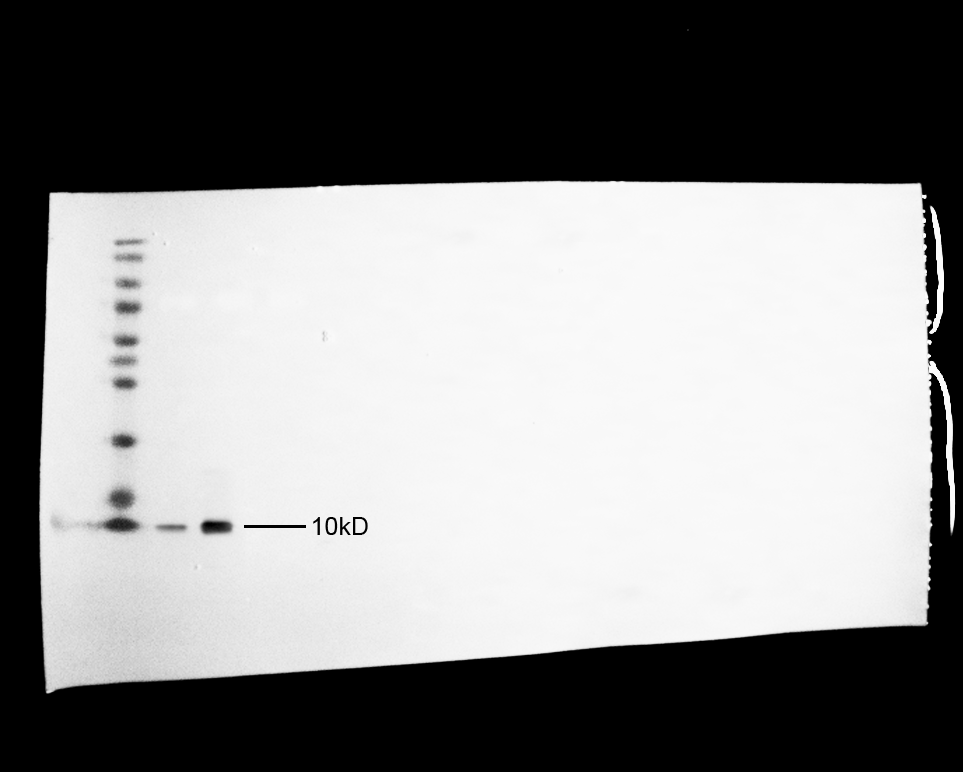 | 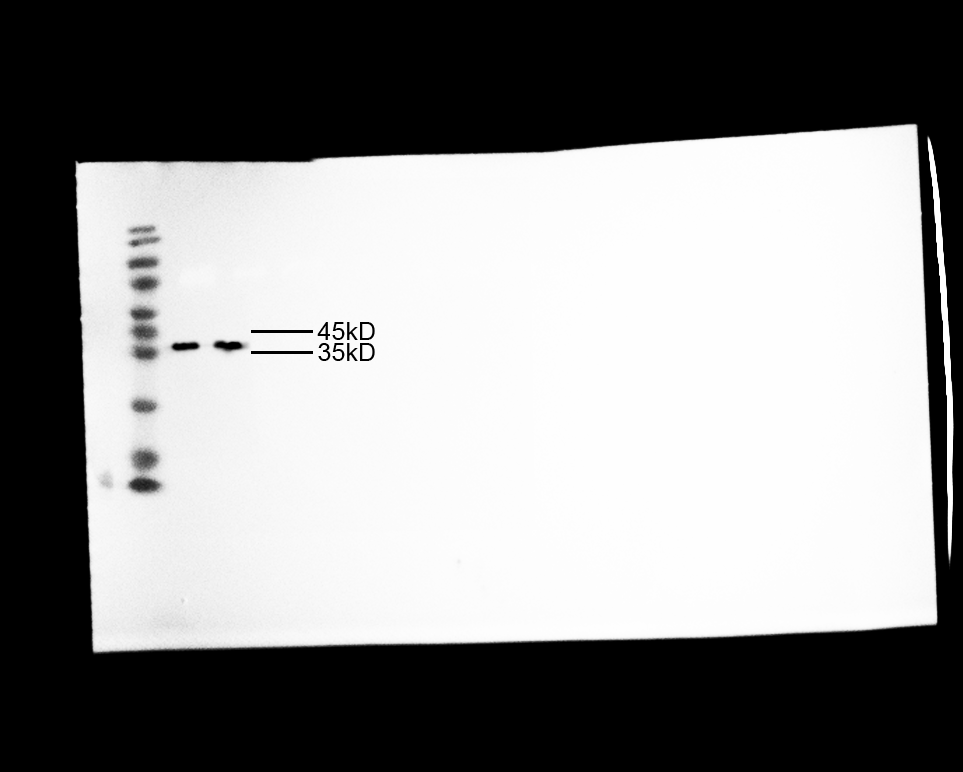 |
| --- | --- |
| 1.Aβ1-42 | 2.GAPDH |

Figure 2C

| 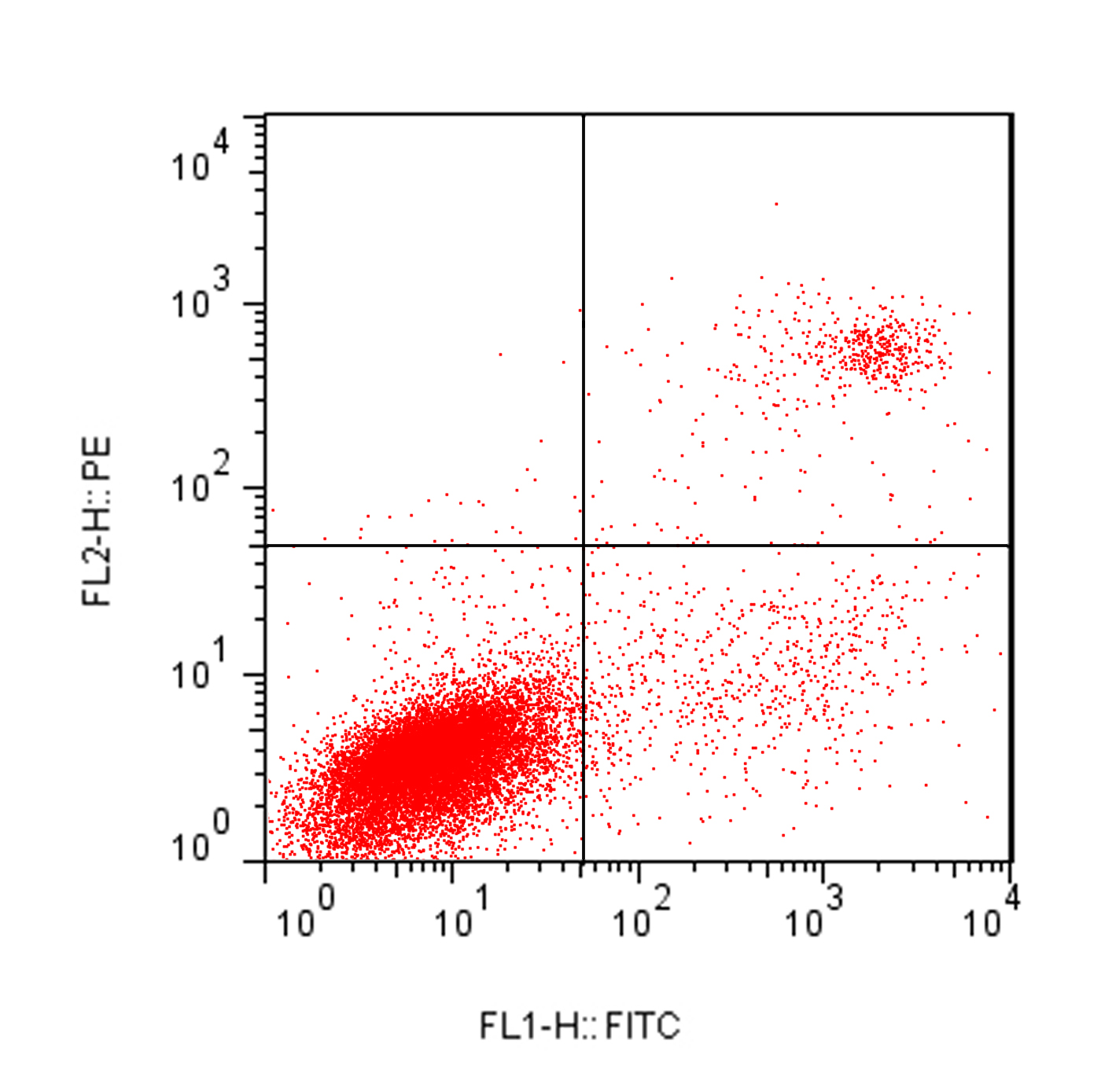 | 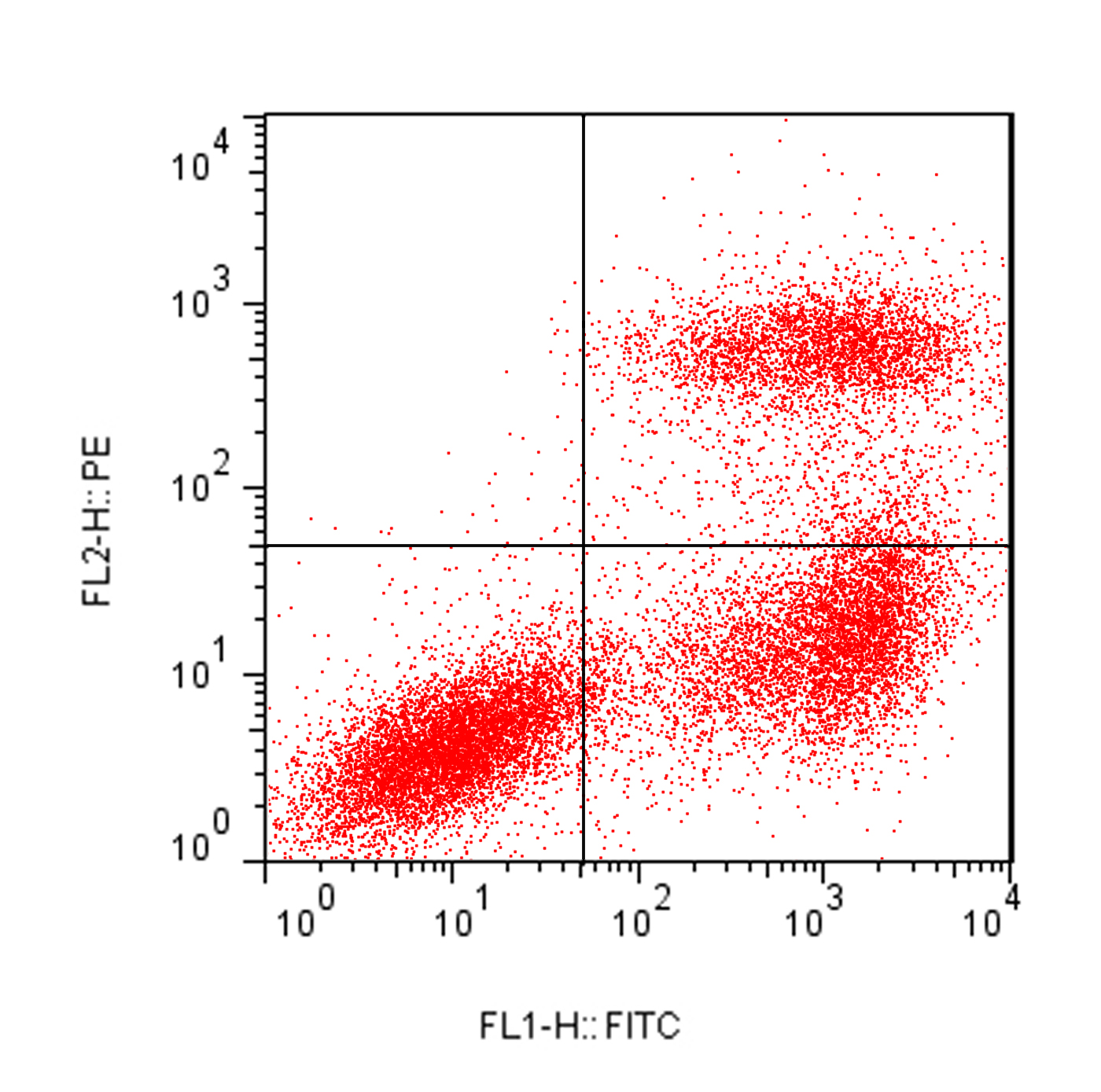 |
| --- | --- |
| 1 | 2 |

Figure 2E

| 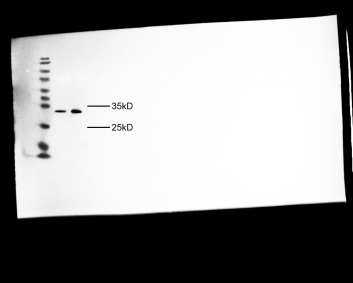 | 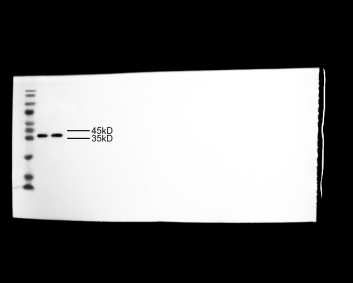 |
| --- | --- |
| 1.NEK7 | 2.GAPDH |

Figure 3C

| 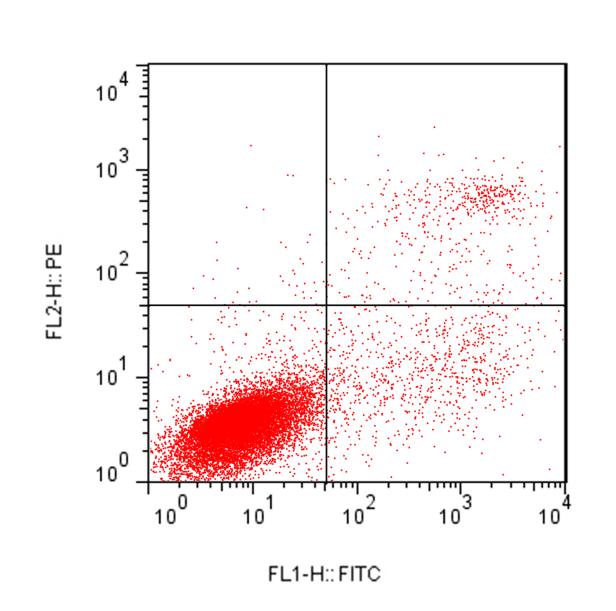 | 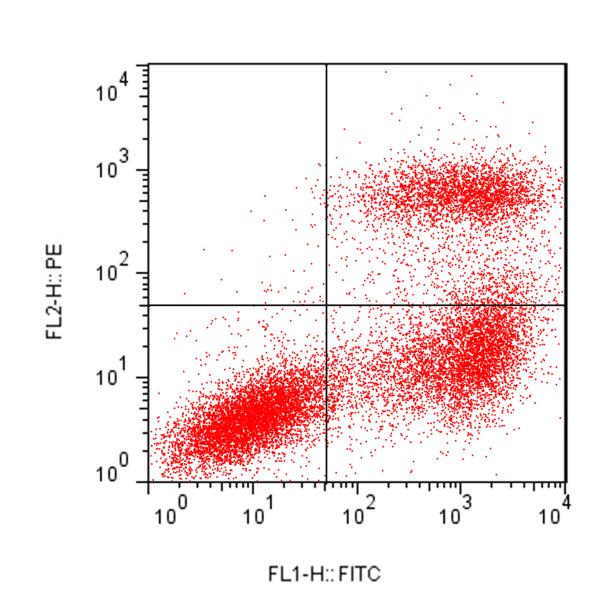 |
| --- | --- |
| 1 | 2 |
| 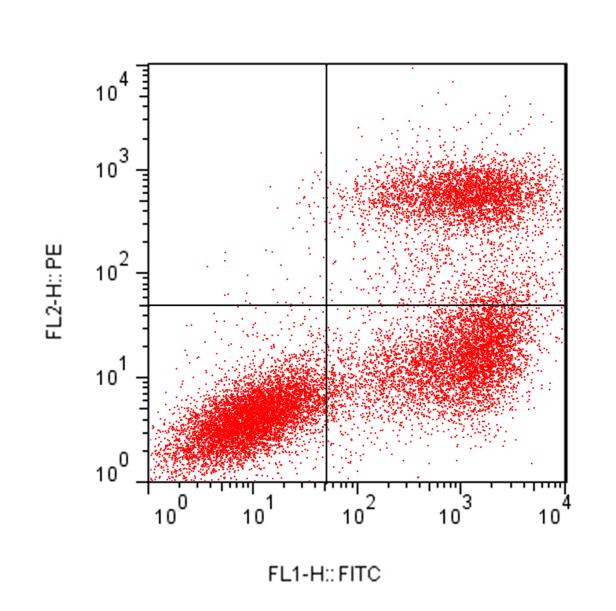 | 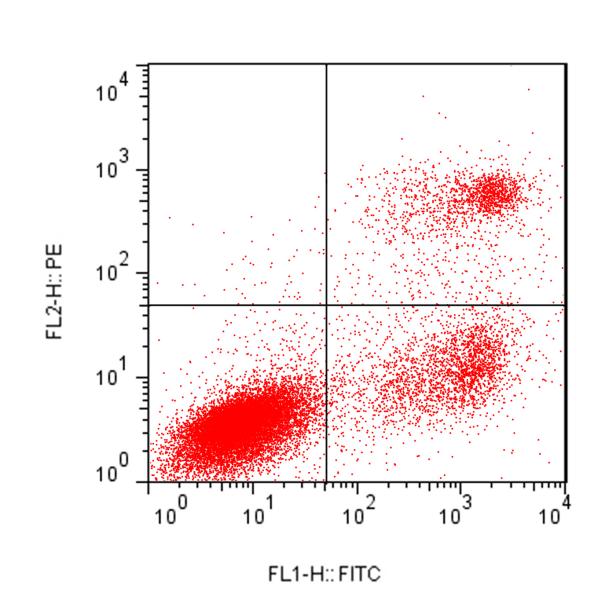 |
| 3 | 4 |

Figure 3F

| 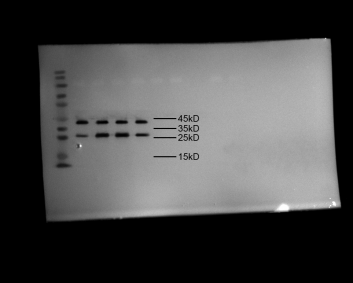 | 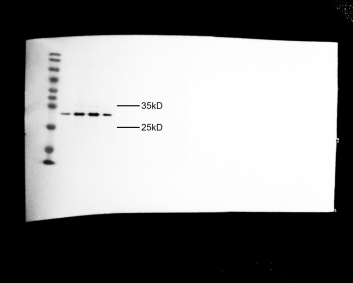 | 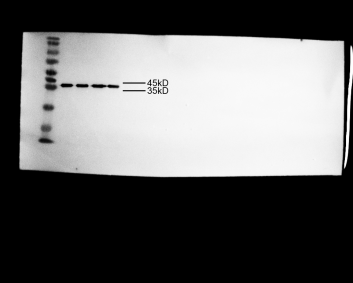 |
| --- | --- | --- |
| 1.caspase1 | 2.GSDMD-N | 3.GAPDH |

Figure 4A

| 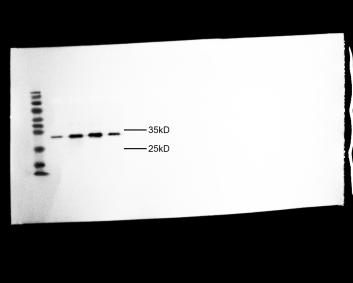 | 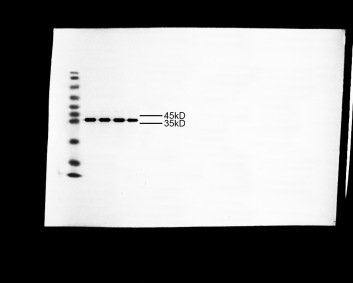 |
| --- | --- |
| 1.NEK7 | 2.GAPDH |

Figure 4B

| 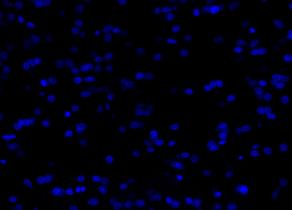 | 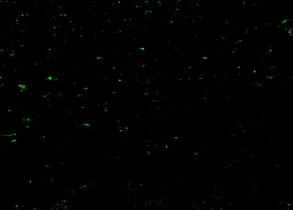 | 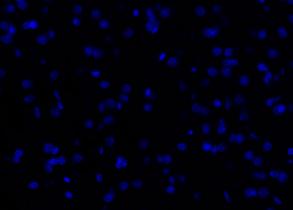 | 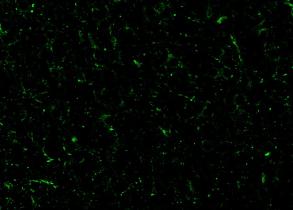 |
| --- | --- | --- | --- |
| 1(1) | 1(2) | 2(1) | 2(2) |
| 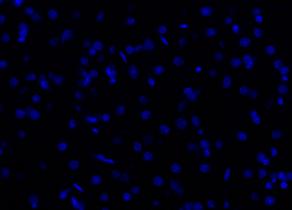 | 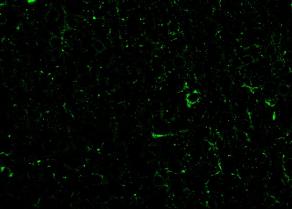 | 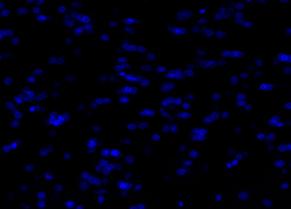 | 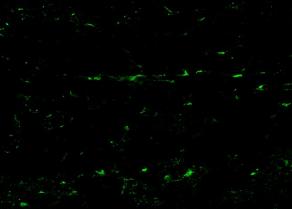 |
| 3(1) | 3(2) | 4(1) | 4(2) |

Figure 5A

| 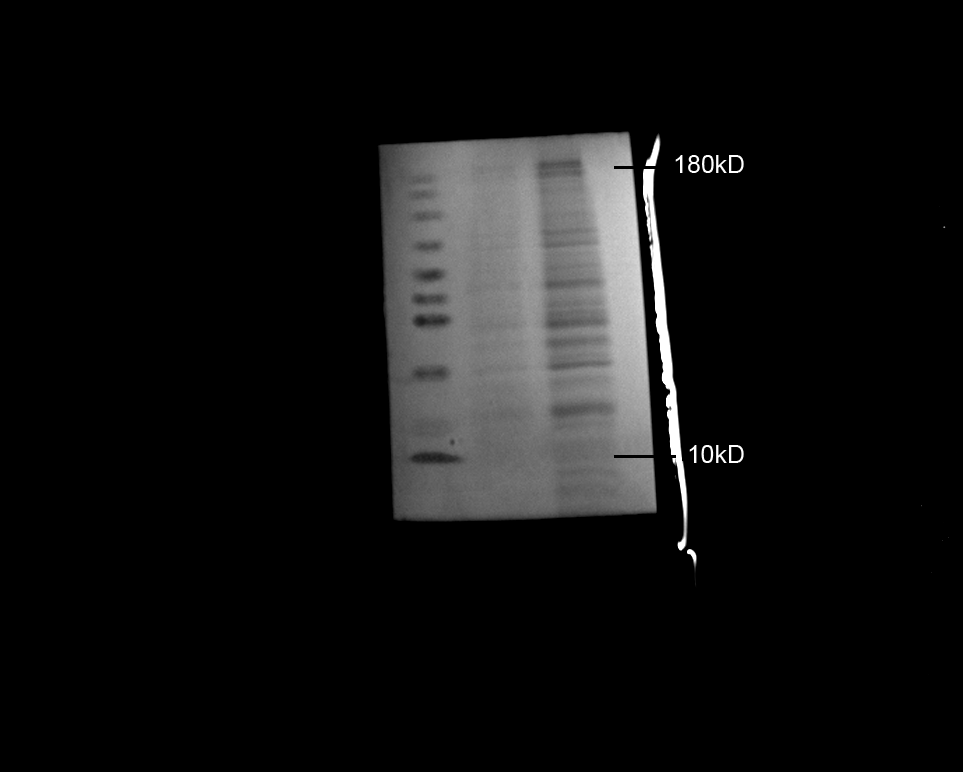 | 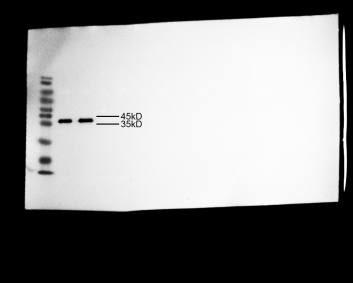 |
| --- | --- |
| 1.Pan-kla | 2.GAPDH |
| 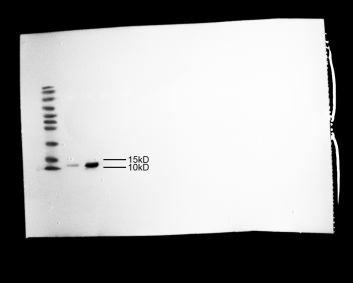 | 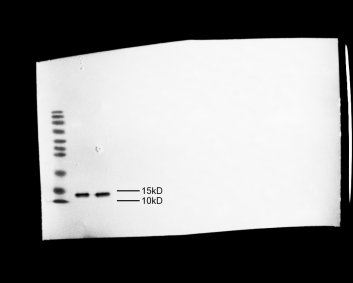 |
| 3.H4K12la | 4.H4 |

Figure 5F

| 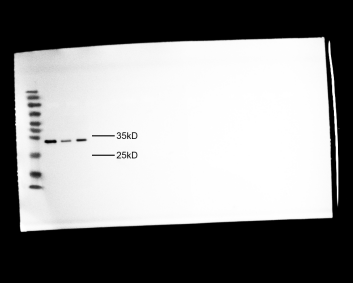 | 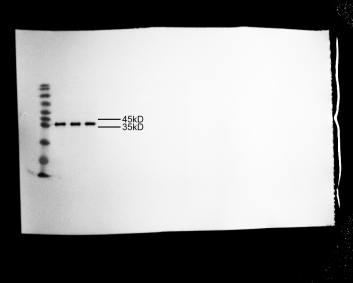 |
| --- | --- |
| 1.NEK7 | 2.GAPDH |
